# Combined effects of bacterial vaginal colonization and HIV on pregnancy outcomes: A Systematic Review and Meta-analysis

**DOI:** 10.21203/rs.3.rs-10307543/v1

**Published:** 2026-08-27

**Authors:** Dismas Matovelo, Quinn Goddard, Paul Sabuni, Benson Kidenya, Jennifer Downs, Moke Magoma, Jeremiah Seni, Kathleen Helen Chaput

**Affiliations:** 1 Catholic University of Health and Allied Sciences, Weill Bugando School of Medicine, Mwanza, Tanzania.; 2 University of Calgary, Cumming School of Medicine, Calgary, Canada; 3 Center for Global Health, Weill Cornell Medicine, New York, USA; 4 Engender Health, Dar es Salaam, Tanzania

**Keywords:** vaginal bacterial colonization, HIV, adverse pregnancy outcomes, preterm birth, low birth weight

## Abstract

The independent effects of HIV and bacterial vaginal colonization on adverse pregnancy outcomes are well established and may be exacerbated by increasing antimicrobial resistance (AMR). However, their combined association with pregnancy outcomes has not been comprehensively evaluated. We conducted a systematic review and meta-analysis to assess the joint effects of HIV and vaginal bacterial colonization on birth weight, preterm birth (PTB), and AMR. MEDLINE, EMBASE, CINAHL, Scopus, Web of Science, Cochrane Library, African Journals Online, and PubMed were searched for observational studies published through December 31, 2023. Eligible studies reported vaginal bacterial colonization stratified by HIV status and pregnancy outcomes. Study quality was assessed using the Newcastle–Ottawa Scale, and pooled odds ratios (ORs) were estimated using random-effects models. Thirteen studies involving 5,807 pregnant women were included. The pooled prevalence of bacterial vaginal colonization was 26% (95% CI: 17.3–37.4%; I^2^= 97.4%). No significant association was observed between HIV-associated bacterial colonization and birth weight (OR= 1.20, 95% CI: 0.08–9.03; p= 0.88). Although the pooled estimate suggested increased odds of PTB, the association did not reach statistical significance (OR= 2.64, 95% CI: 0.99–6.96; p= 0.05). HIV infection was not significantly associated with bacterial vaginal colonization (OR= 1.08, 95% CI 0.40–2.92), and no significant differences in AMR patterns were identified between women with and without HIV. Current evidence does not support a significant combined effect of HIV and bacterial vaginal colonization on birth weight or PTB. Larger, methodologically robust prospective studies are needed to clarify these relationships.

## INTRODUCTION

Lower genital tract bacterial colonization (LGTBC) is usually polymicrobial, affecting up to 50% of women during pregnancy, intrapartum, and postpartum, and varying across trimesters due to hormonal changes, host genetics, behaviour, nutrition, and environment(1–3). Vaginal bacterial composition during gestation typically remains relatively stable among pregnant women not living with HIV(4). In contrast, increased pro-inflammatory responses and the release of HIV-associated cytokines disrupt the integrity of the vaginal mucosal epithelium. This loss in integrity results in the vaginal mucosa of HIV-infected pregnant women being characterized by a diverse microbiota predominantly comprised of anaerobic species(3). Accordingly, studies have reported a higher prevalence of LGTBC among pregnant women with HIV(5). However, others report no differences(6–8). Estimates of the burden of LGTBC during pregnancy, regardless of HIV status, vary widely, with prevalences from 1.5–73.5%(9, 10).

Over the past decade, it has become clear that although a healthy placenta has no microbiome, it is still susceptible to pathogenic colonization, commonly GBS(11). Placental bacterial colonization can affect placental development and function, impacting fetal growth and development. Disrupted placental function can lead to SGA, IUGR, preterm labour and birth, rupture of membranes (ROM), chorioamnionitis, neonatal sepsis, and low birthweight (LBW)(11, 12). These factors contribute to a series of short and long-term adverse effects, such as neonatal sepsis and delayed child developmental milestones(11, 12). The impact of bacterial colonization in pregnancy is disproportionately felt in Low-Middle Income Countries (LMICs) while optimizing surveillance and treatment in high-income countries (HICs) has recently significantly reduced occurrence of LGTBC in pregnancy(13), such routine surveillance is lacking in most LMICs. The rapidly escalating burden of antimicrobial resistance (AMR) has complicated empirical treatment in LMICs (14, 15). The burden of HIV is also disproportionately felt in LMICs, with approximately 70% of people living with HIV in sub-Saharan Africa(16, 17).

LBW and preterm birth (PTB) globally affected an estimated 19.8 and 13.4 million births in 2020, respectively, with increased mortality risks, particularly in the neonatal period, and long-term adverse health outcomes throughout life(12, 18, 19). However, there is a paucity of evidence on the association between HIV, LGTBC and birth outcomes. To bridge this knowledge gap, we performed a systematic review and meta-analysis to quantitatively assess the prevalence of LGTBC and their association with pregnancy outcomes in women living with HIV and those without. We hypothesize that concurrent HIV and LGTBC will increase the odds of unfavourable pregnancy outcomes (e.g., PTB, LBW).

## METHODS

### Objective

The current review examined the combined effects of LGTBC and HIV during pregnancy on birthweight and PTB, intrauterine growth restriction (IUGR), and small for gestation age (SGA). As a secondary outcome, we assessed the prevalence of AMR among HIV-infected vs -uninfected pregnant women. This review study was guided by the Preferred Reporting Items for Systematic Review and Meta-Analysis (PRISMA) protocols, available in the supplementary material of this article (Supplemenatary Table S1: PRISMA 2020 checklist)(20) and was registered with PROSPERO(21) (CRD42023485123).

### Search Strategy

A comprehensive search strategy was developed in consultation with a health sciences research librarian and reviewed by two perinatal researchers; it is available in the supplementary material of this article (Supplementary Note S3: Literature Search Strategy). Our controlled vocabulary search included the following exploded keywords: *Vaginitis, “Genital Diseases, Female”, “Reproductive Tract Infections”, vagina, bacteria, Escherichia coli, “Klebsiella pneumoniae”, “streptococcal infections”, HIV, HIV Seropositivity, HIV Infections, HIV Seronegativity, Pregnancy, Pregnant Women, Pregnancy Trimester, Third, and pregnancy outcome*.

We applied the search strategy in MEDLINE (Ovid), EMBASE (Ovid), CINAHL, Scopus, Web of Science, the Cochrane Library, African Journals Online, and PubMed to identify studies up to December 31, 2023, with no language restrictions. Web of Science, Google and Google Scholar were used to search for grey literature. We modified indexing terms, such as Medical Subject Headings (MeSH) or field tags, for each database and linked terms using the Boolean operators “AND” and “OR”. We hand-searched by scanning references, “cited-by”, and “similar articles” lists for eligible studies. No filters were applied.

### Eligibility criteria

We included original quantitative observational studies published in peer-reviewed journals that reported on LGTBC and HIV status in the context of our target pregnancy outcomes (LBW, PTB, IUGR, SGA) and/or AMR of bacterial isolates obtained during pregnancy. Specifically, eligible studies involved pregnant women of any age tested for LGTBC regardless of the gestation age sampled antenatally before delivery.

Studies must have had an exposure arm with PWLWH and a control arm without HIV. Studies must have measured birth weight and/or PTB as a pregnancy outcome to document the differences in associations between LGTBC and birth weight among PWLWH and those without HIV. The outcomes included aggregate counts of PTB (<37 weeks’ completed gestation) and low birth weight (<2500g). We included grey literature and published abstracts in the narrative summary of the review.

### Study selection

We used Covidence to collate our initial search results and remove duplicates before screening. Two independent authors (DM and QG) screened titles and abstracts and assigned a ‘yes’, ‘no’, or ‘maybe’ to their eligibility for inclusion. Studies marked as “yes” or “maybe” underwent a full-text review, during which reasons for exclusion were documented. A third author (KHC) resolved any conflicts in decision-making.

### Data Extraction and Critical Appraisal

DM and QG independently extracted data using Covidence (exported into Microsoft Excel 2023). We extracted: a) first author’s name, b) publication year, c) region/country, d) study design, e) sample size, f) type of LGTBC sample collected, and g) microorganisms involved. To assess the target outcomes, we extracted the number of individuals with the unfavourable birth outcome of interest (e.g., PTB) and the favourable converse (e.g., term birth), stratified by HIV and LGTBC status (regardless of microorganism, given the small number of studies). Discrepancies were resolved by a third individual (KHC). If desired data were unavailable for extraction, we contacted authors thrice between January and June 2024 to obtain missing data points. Studies were excluded from the meta-analysis if the authors were unreachable or unable to share the required data. Non-English studies were evaluated using Google Translate and then extracted in consultation with a native speaker however, no non-English studies qualified for extraction.

### Quality assessment

We used the Newcastle-Ottawa Scale (NOS) because all eligible studies were observational (22). The NOS scale consists of eight sections that evaluate an article based on selection criteria, comparability, exposure assessment, and outcomes. The first (DM) and second (QG) authors performed the quality assessment, which was verified by the senior author (KHC). Each included study received a score corresponding to the number of quality criteria satisfied and was categorized as low (0–5), medium (6–7), and high (8–10) quality. Because all eligible studies scored ≥7, all were included.

### Data analysis

A narrative analysis summarizes the characteristics of the included studies. We quantified the pooled joint association of HIV status and LGTBC with pregnancy outcomes, using random-effects maximum likelihood forest plots to calculate log-odds ratios (OR) and their corresponding 95% confidence intervals. We applied a continuity correction of 0.5 for insufficient cell counts (23). We assessed heterogeneity using Cochrane’s Q value and Higgins I-squared (I^2^), with I2 values of 0–40% considered low, 50–70% moderate, and >70% high (22). We conducted all analyses using Stata version 18.0.

Subgroup analyses were planned a priori according to bacterial species, study design, geographical region, and diagnostic method. However, these analyses were not undertaken because the number of studies contributing to each subgroup was insufficient to generate reliable pooled estimates. Furthermore, substantial clinical and methodological heterogeneity, along with incomplete reporting of subgroup-specific data, limited meaningful comparisons.

## Results

**Figure 1:**
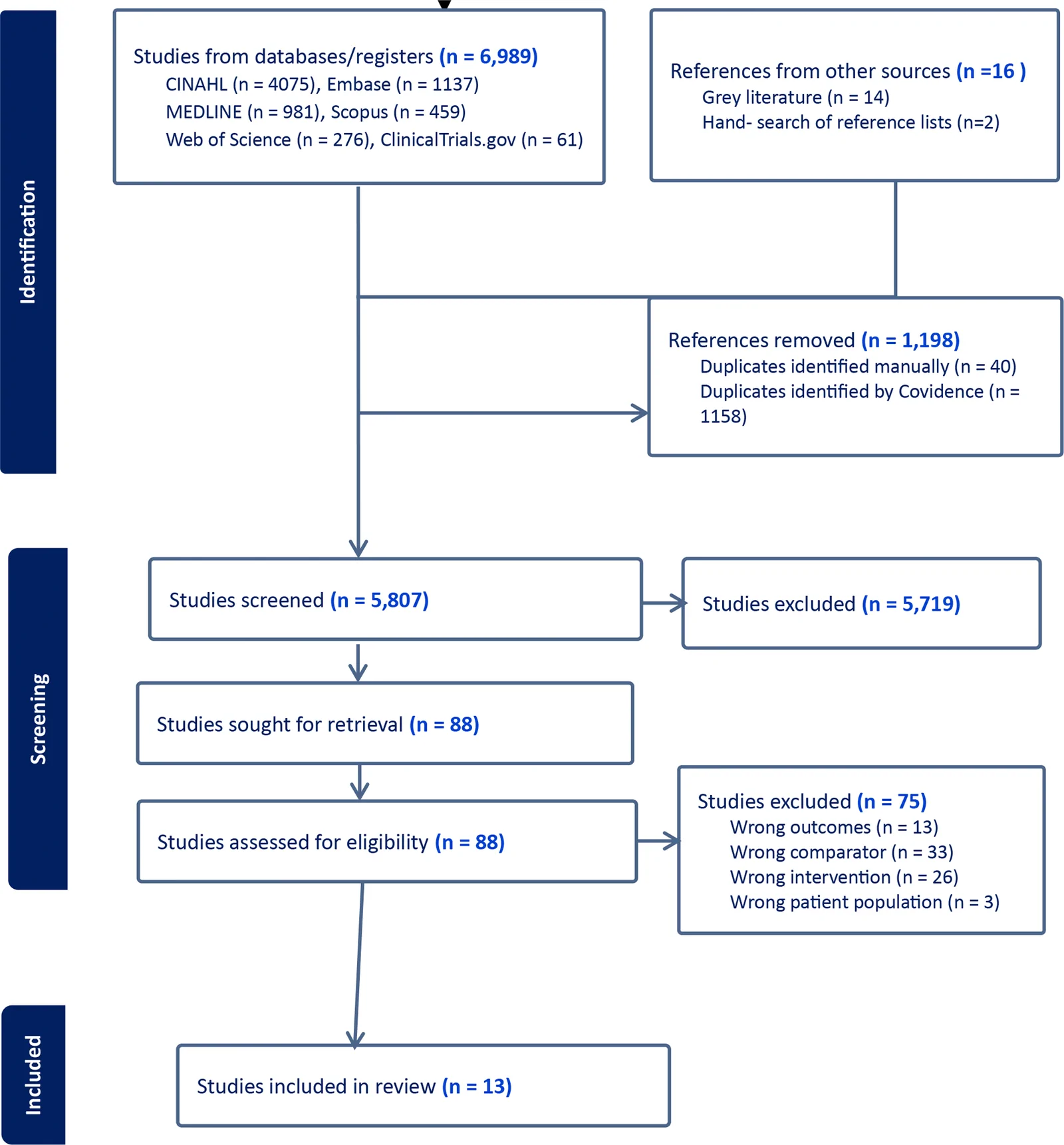
PRISMA flowchart diagram of the study selection.

We identified 5,805 identified studies (initially 7,005, 1,198 duplicates removed; Medline=981, Embase=1,137, Web of Science= 276, CINAHL=4,075, Scopus=459, Cochrane=61, and grey literature (ProQuest)=14). DM and QG screened titles and abstracts with high inter-rater agreement (proportionate agreement=0.97, Cohen’s kappa 0.75), deeming 5,719 irrelevant. Two additional studies were identified through hand-searching reference lists, leaving 86 studies for full-text assessment. From that, 13 studies were identified for inclusion in the systematic review (Figure 1, Table 1).

All identified studies were rated 7 or higher on the NOS by the first and second authors. No studies were excluded based on quality (Table 2). Since most included studies did not report all data required for meta-analysis, we contacted the authors of all studies to request additional data. All included studies were conducted between 2006 and 2023. Four were cross-sectional, seven were cohort, and two were case-control studies. All except three were conducted in Africa. We found no other published systematic reviews on this topic.

The overall sample size of all included studies was 6,073 participants, with individual study sample sizes ranging from 75 to 1,857. The prevalence of LGTBC ranged from 3.3% to 69.3% (Table 1). Testing and diagnostic criteria for LGTBC varied (Table 3).

## Narrative review

All identified studies measured our variables of interest; however, very few studies stratified data according to HIV status and bacterial colonization simultaneously. This narrative review summarizes only the results reported in the studies, and the data included in our meta-analysis were largely obtained directly from the authors upon request.

Studies varied according to the association between LGTBC, HIV, and LBW, while Makinde *et al*(32) and Gudza-Mugabe *et al*(26) found no association, Nyemba *et al*(25) found that LGTBC (here, *C. trachomatis* and *N. gonorrhoea*) at the first antenatal care (ANC) visit in individuals with HIV led to an increased risk of adverse birth outcomes.

Results for PTB were more consistent: Nyemba *et al. [25], Smullin et al. [30], and Short et al.* (31) all found an elevated risk of PTB in the combined association between LGTBC and HIV status. However, Gudza-Mugabe *et al*(26) and Price *et al*(28) found that individuals with HIV were more likely to give birth prematurely, regardless of vaginal microbiota, suggesting LGTBC did not modify the relationship between HIV status and PTB. Ravindran *et al*(34) found that incident HIV, as well as LGTBC (here, *C. trachomatis* and *N. gonorrhoeae)*, all independently increased the risk of PTB. El Beitune *et al*(36) found no significant difference in birth outcomes by maternal HIV status. Benedetto *et al*(37) found that cervicovaginal colonization (BV, *C. trachomatis*) increased the chance of PTB. Foessleitner *et al*(38) found that PTB birth was equally likely regardless of HIV status. Govender *et al*(39) analyzed the prevalence of genital mycoplasmas, ureaplasmas, and *Chlamydia trachomatis* to HIV status, finding no association between HIV status and LGTBC, as well as preterm delivery and LGTBC.

Several additional studies examined our variables of interest, but we could not include them due to lack of access to the original data. Zenebe *et al*(40) found that mean birthweight was higher in babies born to HIV-uninfected women. Joachim *et al*(41) found no significant association between infant birthweight and GBS carriage. Seale *et al*(42) found that women with GBS had an equal likelihood of very PTB and very LBW. Madrid *et al*(24) found that mothers with HIV were at no higher risk of *GBS* colonization. Morgan *et al*(33) found that HIV-positive individuals had a higher chance of PTB but that the likelihood of GBS colonization did not differ by HIV status.

Only two studies analyzed SGA: while Temmerman *et al*(43) found mothers with HIV were more likely to give birth to SGA infants, Gudza-Mugabe *et al*(26) found no difference in SGA by maternal HIV status. None of the included studies analyzed IUGR. It is important to note that bacterial species varied across studies (i.e., *GBS*, *C. trachomatis, N. gonorrhoeae*), which may explain the differing results.

### Meta-Analysis

#### Primary Outcomes:

Five studies examined LBW as an outcome, with an overall sample of 3,520 pregnant individuals, 784 (22.2%) of whom had LGTBC and 1,284 (36.4%) had HIV. Eight studies examined PTB, with a total sample of 5,164 pregnant individuals, 1,356 (26.3%) of whom had LGTBC and 1,372 (26.6%) had HIV. We had insufficient data to generate meaningful pooled estimates for IUGR or SGA.

#### LBW:

**Figure 2:**
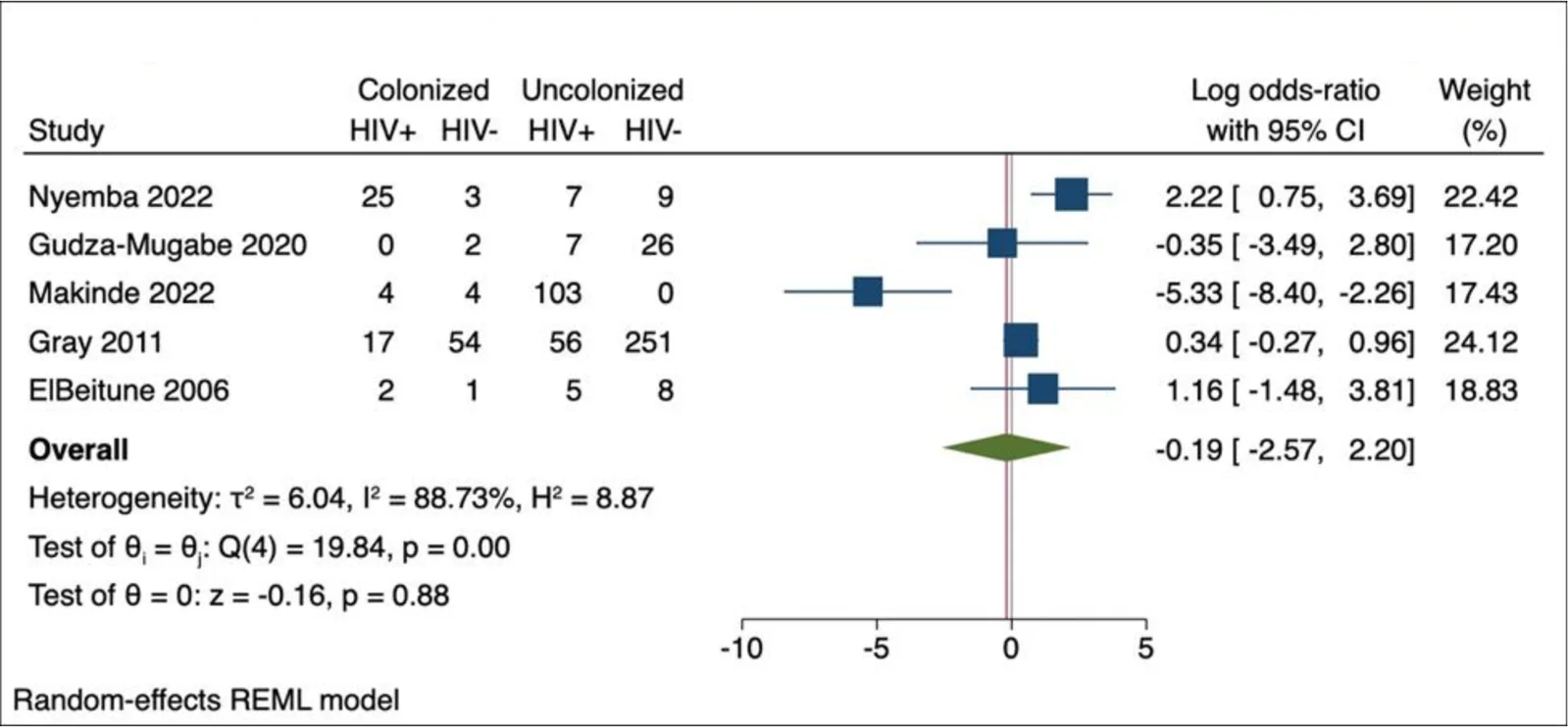
Joint association of HIV status and bacterial colonization with birthweight

We observed no significant joint association between HIV status and LGTBC on low birth weight (OR=1.21, *p*=0.88). The high heterogeneity between studies (*I*^*2*^=88.73%; *Q*=19.84, *p*=0.00) indicates low reliability for the pooled estimate (Figure 2).

#### PTB:

**Figure 3:**
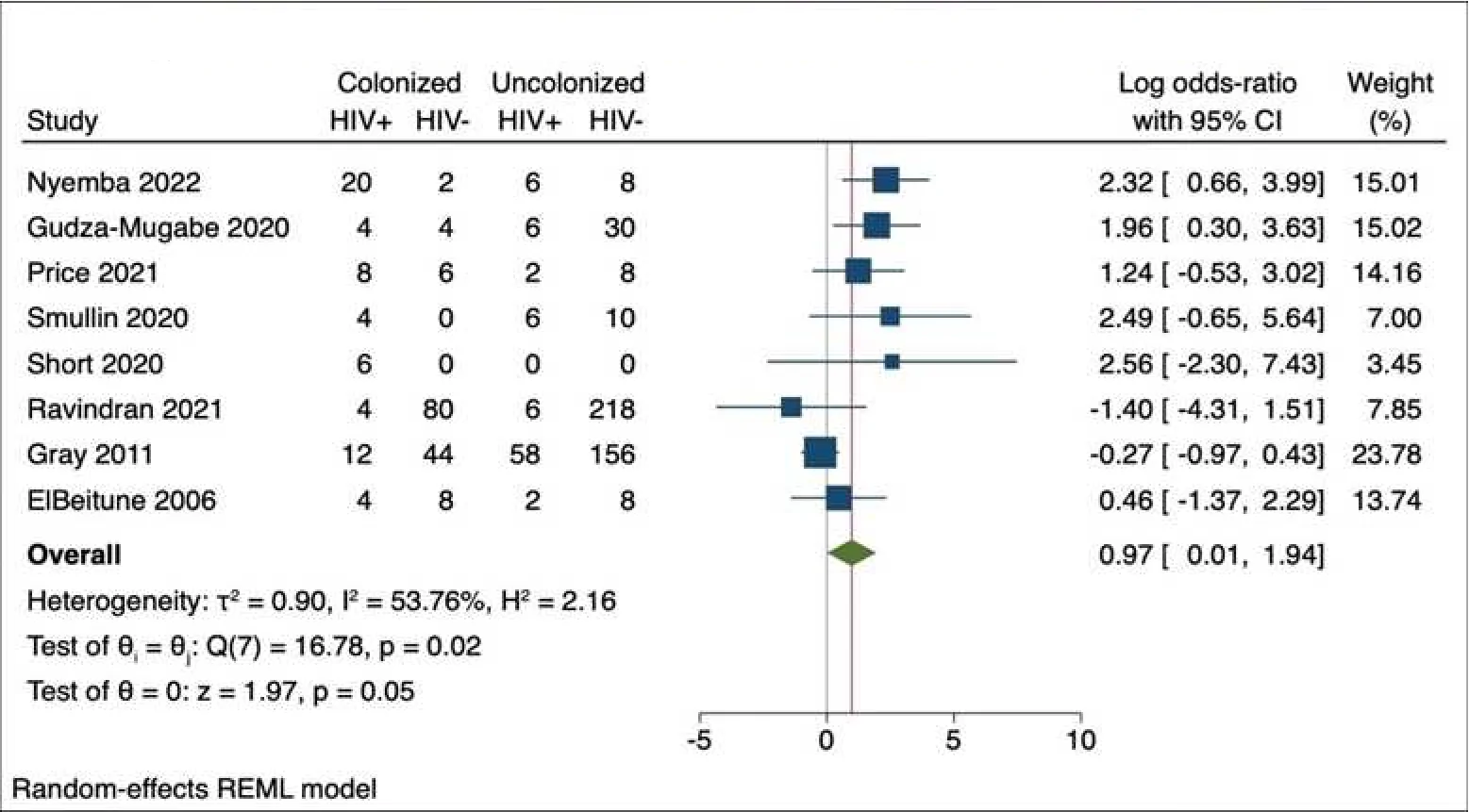
Joint association of HIV status and bacterial colonization with PTB

Overall, we found suggestive evidence of increased odds of PTB among individuals with HIV and LGTBC (OR: 2.64, *p*=0.05) compared with those without. Moderate heterogeneity was observed among the included studies (I2=53.76%; Q=16.78, p=0.02), indicating moderate reliability of the estimates (Figure 3). The methods for calculating gestational age varied (last menstrual period (25, 26, 35), ultrasound dating (28), fundal height (34), or unspecified(30, 31)), potentially contributing to heterogeneity.

### Heterogeneity and Publication Bias

Since fewer than ten studies were included in the meta-analysis, publication bias and sources of heterogeneity were not formally assessed using funnel plots or regression tests, as these methods are unreliable for such a small number of studies due to underpowering. Therefore, small-study effects cannot be excluded.

#### Secondary Outcome:

In assessing AMR, we found that six different types of bacterial isolates were examined in the included studies, with the highest pooled prevalence being bacterial vaginosis (33.9%) (Table 3). All studies testing AMR evaluated *GBS*, thereby facilitating meaningful pooled estimates of the association between HIV and GBS with respect to AMR.

**Figure 4:**
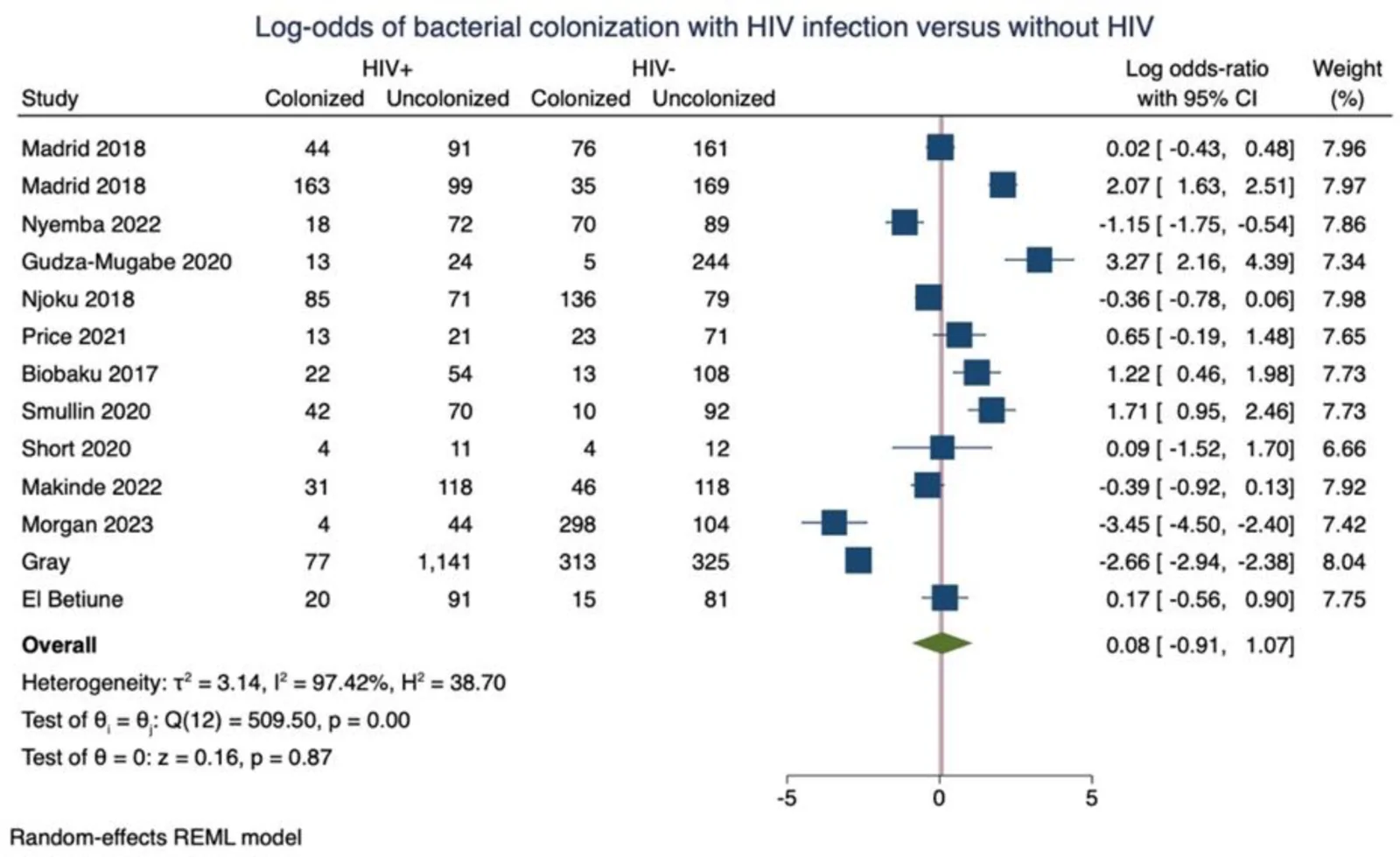
Association between HIV status and Bacterial Colonization

**Figure 5:**
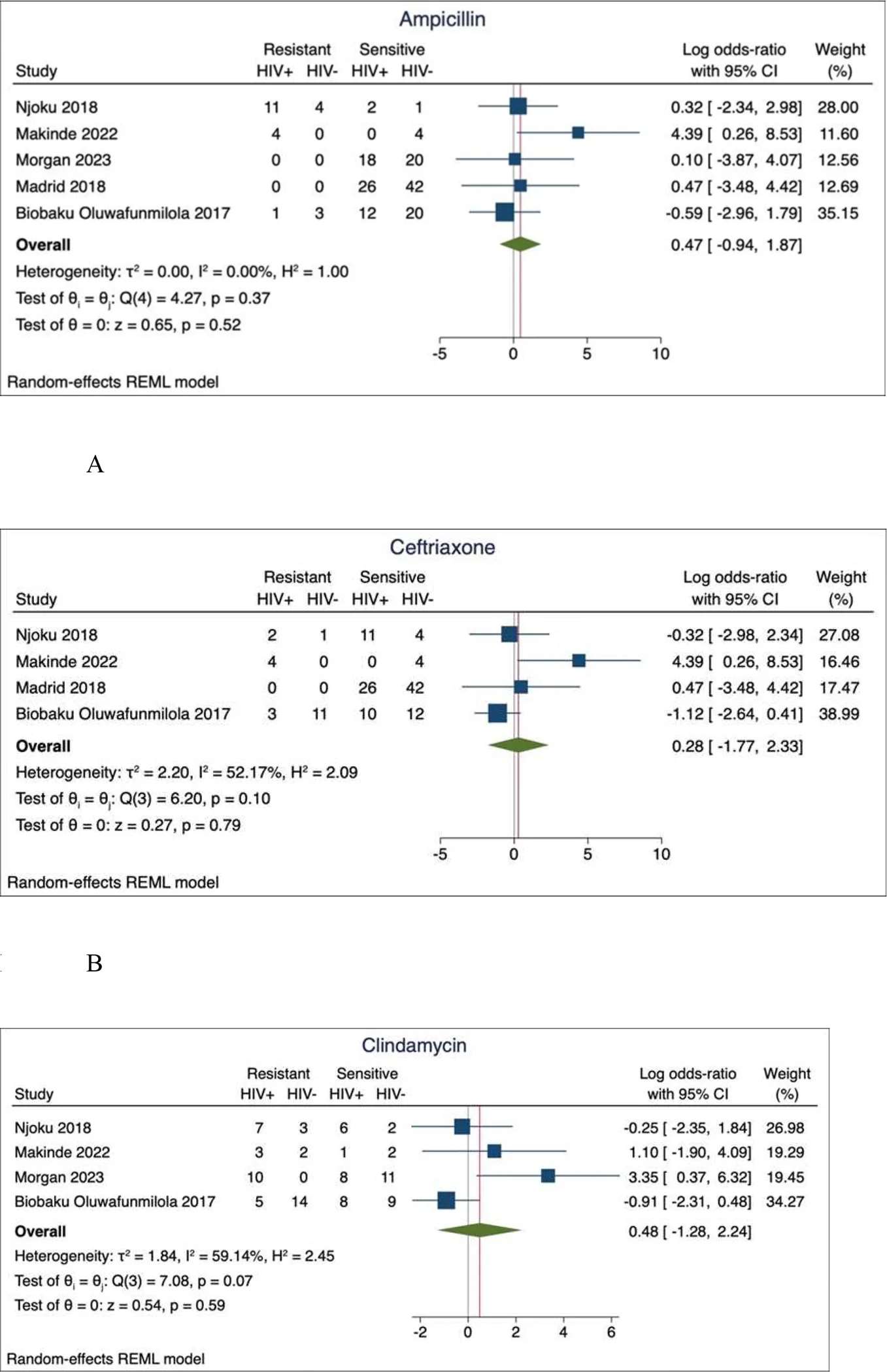

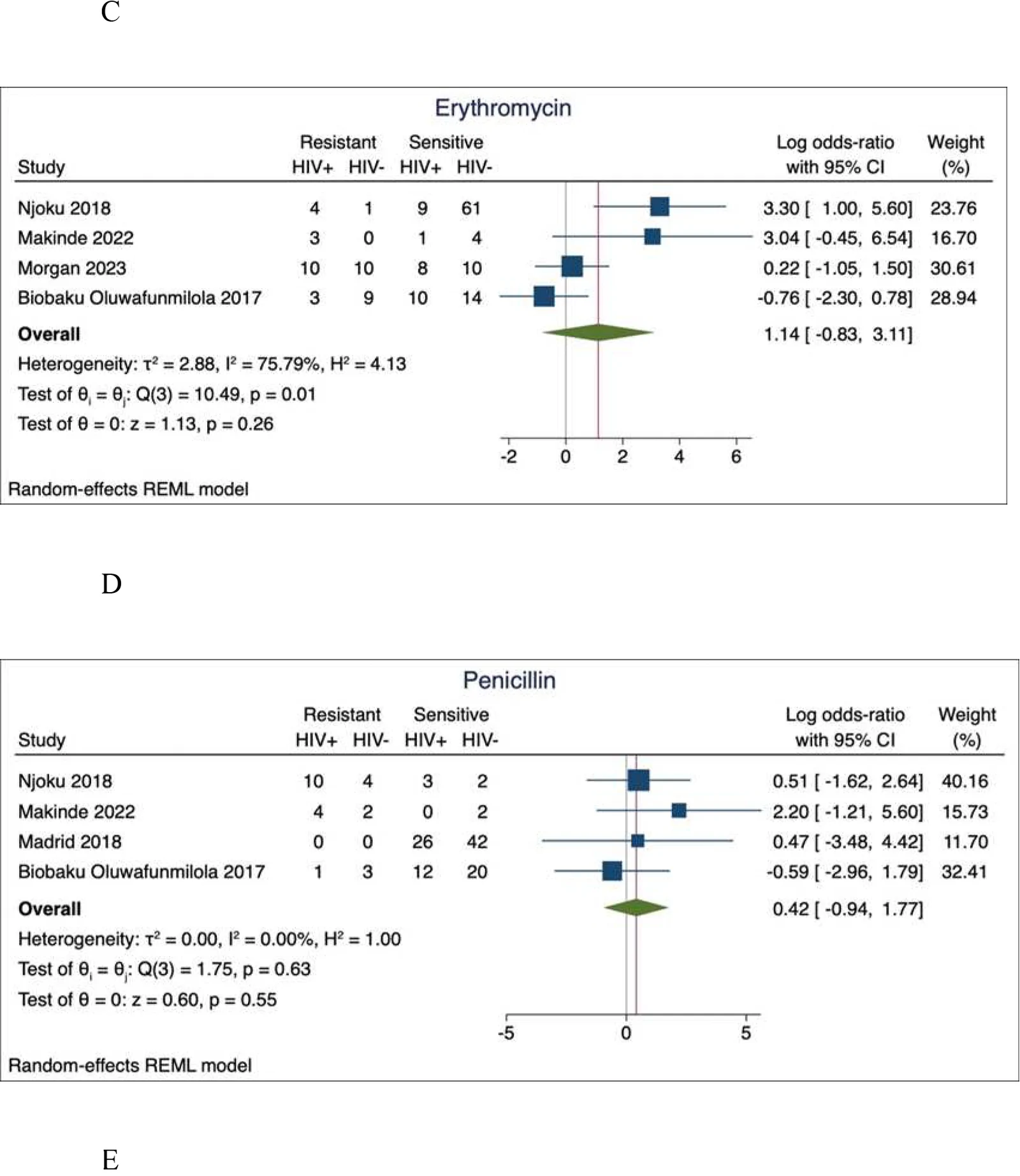


There was no significant association between HIV status and LGTBC (OR=1.08) (Figure 4). Likewise, we found no significant difference in log odds of AMR between HIV-positive and HIV-negative individuals for each antibiotic examined (Ampicillin: OR=1.60, Ceftriaxone: OR=1.32, Clindamycin: OR=1.61, Erythromycin: OR=3.12, Penicillin: OR=1.42). Heterogeneity was low to moderate for most antibiotics, except for erythromycin (I^2^=75.79) (Figure 5:A–E). We could not pool proportions of AMR across antibiotic types due to aggregate reporting of AMR and lack of individual participant data.

## Discussion

### Main findings

From the 13 studies included, we found the odds of PTB among those with HIV and LGTBC were 2.64 times greater than those without HIV or colonization, albeit with an observed trend toward increased odds (OR:2.64, p=0.05). Although the pooled estimate suggested increased odds of PTB among women with both HIV and LGTBC, the association did not reach conventional statistical significance. No significant combined effect between HIV and vaginal bacterial colonization on LBW was observed (OR:1.21, *p*=0.88). We found a pooled prevalence of bacterial colonization of 26% (95% CI: 17.3%, 37.4%) among pregnant women, but no significant difference in colonization by HIV status (OR: 1.08; 95% CI: −0.91, 1.07). Similarly, no significant differences in AMR were found between those living with HIV and those without for ampicillin, ceftriaxone, clindamycin, erythromycin, or penicillin.

### Strengths

We found no previous systematic review or meta-analysis that assessed the potential joint relationship between HIV status, LGTBC and LBW. One of the strengths of this study is the inclusion of studies examining direct vaginal colonization. Previous studies evaluated rectal colonization only, meaning previous evidence may include pathogens not present in the lower genital tract. Thus, it may not affect pregnancy to the same extent (44, 45). Although our review’s large pooled sample size (*n* = 6,073) and the high quality of the included studies are strengths, the limited number of studies, coupled with the moderate-to-high heterogeneity of estimates, limits the reliability of the findings.

### Limitations

Although this systematic review and meta-analysis present up-to-date global evidence on the prevalence of vaginal bacterial colonization and its association with pregnancy outcomes, it is not without limitations. Sensitivity analyses were not performed because of the limited number of studies contributing to each meta-analysis, which would have resulted in unstable estimates following study exclusion. Furthermore, all included studies were of moderate to high methodological quality, reducing the value of excluding studies based on quality alone. Each pathogen represents a biologically distinct exposure. Removing a single study could effectively eliminate an entire pathogen category, leading to sensitivity analyses that reflect changes in exposure type rather than in study quality. Future meta-analyses incorporating a larger body of evidence should undertake sensitivity, subgroup, and meta-regression analyses to better investigate sources of heterogeneity.

Only 5 studies reported on PWLWH receiving ART and their CD4 counts; none reported on viral load, all variables likely to influence colonization and PTB. The majority of the included studies are from Africa, where the prevalence of HIV is high. Comparatively, there is a lack of studies from Asia, Europe, and North and South America. Therefore, generalizability is limited to African populations, as the overall sample does not represent a global population.

Further, most included studies recruited participants and/or laboratory samples through hospitals or from mothers who presented for routine ANC or delivery at tertiary hospitals. This could skew the estimated prevalence towards urban settings and underestimate the associations in rural areas or in smaller local health centres serving individuals with more vulnerable socioeconomic status. The risk of AMR likely differs between these tertiary hospitals and rural settings (e.g., people seeking care at tertiary hospitals may have greater or more frequent exposure to antibiotics). There may be some publication bias toward studies reporting elevated odds of adverse outcomes. However, our null results suggest this bias is likely minimal.

### Interpretation

Our pooled estimate should be interpreted as representing pathogenic LGTBC collectively rather than the effect of any specific bacterial species in which we observed a trend toward increased odds of PTB. Therefore, even with pooling study results, we may have lacked sufficient statistical power to adequately assess the effect. However, our findings regarding PTB are similar to those from previous reviews evaluating HIV status independently; for instance, Xiao *et al.* found an increased odds of PTB among individuals with HIV compared to those without(46). While one of the studies included in our review found a decreased risk of PTB in PWLWH with colonization, this finding may be attributable to the small sample size of PWLWH in this study (*n*=10), as well as the fact that they did not recruit based on HIV status and only happened to retain cases of HIV acquired during the study period in their originally HIV-negative cohort(34).

Turning to LBW, our null finding differs from previous reviews, which have demonstrated that HIV-exposed neonates are at higher risk for GBS neonatal disease(47) and that LBW is more prevalent in neonates from women living with HIV(46). Therefore, available evidence may have been underpowered to detect the combined effect. Unfortunately, we could not model the pooled effect on gestational age or birthweight outcomes as continuous variables due to data limitations. Future studies should report incidences of both continuous and categorical outcome measures to allow for a more nuanced assessment of this relationship.

We observed moderate to substantial heterogeneity among our included studies. This may be due to different pathogenic bacteria colonizing the lower genital tract, timing of specimen collection and obstetric risk profiles. Additionally, some methodological differences in the studies included different study designs, diagnostic tests, and definitions of PTB, as well as selection and inclusion criteria such as ART exposure, HIV disease severity, gestational age at sampling/culture, and antibiotic exposure. Further heterogeneity may stem from differences in population characteristics, study designs and geographical locations. However, we were underpowered to perform any sensitivity analyses to evaluate sources of heterogeneity.

We also found that the overall odds of bacterial colonization did not differ significantly between those living with HIV and those without. This finding is congruent with a review by Cools *et al.,* which showed no difference between HIV status and rectovaginal colonization(47). However, few of these studies were designed to assess the association between HIV status and bacterial colonization as a primary outcome. Future prospective cohort studies with strong selection and recruitment strategies, along with adequate confounding control in the analysis, are necessary to confirm whether HIV status impacts the odds of bacterial colonization in pregnancy.

While we found no significant difference in AMR by HIV status, the small sample sizes and the lack of individual-level data meant we could not pool across antibiotic types, which limited statistical power. Future studies should consider including measures of resistance to one or more antibiotics to enable pooled estimates without the risk of double-counting. A meta-analysis found those with HIV were more likely to be colonized with resistant bacterial strains(48), it remains unclear if similar patterns persist during pregnancy.

The result of this meta-analysis indicates there is a substantial need for adequately powered prospective studies to quantify the magnitude of impact that HIV and LGTBC jointly have on pregnancy outcomes. Further research with large sample sizes, stringent selection criteria, reliable and valid measurement, adequate adjustment for potential confounders, and the assessment of birthweight and gestational age at delivery as continuous outcomes is desirable to strengthen the evidence in this area. Since LGTBC in HICs tends to be predominantly Gram-positive, whereas in LMICs it tends to be predominantly Gram-negative, which is more strongly associated with neonatal sepsis and mortality, this emphasizes the need for further research that considers a geographical context. Further, there is still no global consensus on the routine screening of pregnant women for lower genital tract pathogenic bacteria. Future research in this area would be vital in informing the development of a standardized vaginal bacterial screening during pregnancy that takes HIV status into account, which could act to improve the identification of pathogenic bacterial colonization and potentially reduce any associated adverse outcomes.

## Conclusion

Bacterial colonization is prevalent among pregnant women, but there is insufficient evidence to suggest that HIV and lower genital tract bacterial colonization are jointly associated with birth weight or PTB. Research with large sample sizes, rigorous participant selection, reliable, valid measurement, and adequate adjustment for potential confounders, with birthweight and gestational age as continuous outcomes, is still needed to provide robust evidence.

## Supplementary Material

Supplementary Files

This is a list of supplementary files associated with this preprint. Click to download.

• SupplementaryTablesS1andS3PRISMA2020ChecklistandSearchStrategy.docx

• SupplementaryDataS2StudyDatabase.xlsx

## Figures and Tables

**Table 1:** Characteristics of the included studies

*s/n*	*Author Name*	*Year published*	*Country*	*Study Design*	*Total (N)*	*HIV Status*		*Colonization n (%)*	*95%CI*	*Type of bacteria*
						HIV+ n (%)	HIV- n (%)			
*1*	Madrid., et al (24)	2018	Mozambique	Cohort	320	117(36.6)	203(64.4)	120(37.5)	32.3%-42.9%	*GBS & E. coli*
*2*	Nyemba., et al (25)	2022	South Africa	Cohort	619	486(78.5)	133(21.5)	198(32.0)	27.6%-34.9%	*Chlamydia trachomatis, Neisseria gonorrhoea*
*3*	Gudza-Mugabe., et al (26)	2020	Zimbabwe	Cohort	356	42(11.8)	314(88.2)	88(24.7)	20.4%-29.3%	*Bacterial vaginosis*
*4*	Njoku., et al (27)	2018	Nigeria	Cross-sectional	168	84(50.0)	84(50.0)	18(10.7)	6.4%-15.9%	*Group B Streptococcus*
*5*	Price., et al (28)	2021	Zambia	Cohort	363	85(23.4)	278(76.6)	221(60.9)	55.8%-65.8%	*Bacterial vaginosis*
*6*	Biobaku., et al (29)	2017	Nigeria	Cross-sectional	198	67(33.8)	131(66.2)	36(18.2)	13.1%-23.9%	*Group B Streptococcus*
*7*	Smullin., et al (30)	2020	South Africa	Cohort	197	92(46.7)	105(53.3)	35(17.8)	12.7%-23.4%	*Mycoplasma genitalium*
*8*	Short., et al (31)	2020	United Kingdom	Cohort	75	53(54.6)	22(45.4)	52(69.3)	58.4%-79.3%	*Bacterial vaginosis*
*9*	Makinde., et al (32)	2022	Nigeria	Cross-sectional	244	122(50.0)	122(50.0)	8(3.3)	1.4%-5.9%	*Group B Streptococcus*
*10*	Morgan., et al (33)	2023	United States of America	Case-control	225	75(33.3)	150(66.7)	77(34.2)	28.2%-40.6%	*Group B Streptococcus*
*11*	Ravindran, et al (34)	2021	Kenya	Cohort	1244	10(0.8)	1234 (99.2)	302(24.3)	21.9%-26.7%	*Chlamydia trachomatis, Neisseria gonorrhoea*
*12*	Gray, et al (35)	2011	Malawi	Case-control	1857	402 (21.7)	1454 (78.3)	390 (21.0)	19.2%-22.9%	*Group B Streptococcus*
*13*	El Beitune et al (36)	2006	Brazil	Cross-sectional	207	101 (48.8)	106 (51.1)	35 (16.9)	12.1%-22.3%	*Group B Streptococcus*
***Overall***					**6073**	**1736(28.6)**	**4336(71.4)**	**1580(26.0)**	**17.3%-37.4%**	

**Table 2:** Distribution of vaginal bacterial colonization among pregnant women

*Author name*	*Total (N)*	*Lab Method*	*Bacteria*	*HIV+*	*95% CI*	*HIV-*		
			
				n (%)		n (%)	*95% CI*	NOS
*Madrid (2018)*	120	Culture	*Escherichia coli*	44(36.7)	28.2%-45.5%	76(63.3)	54.5%-71.8%	8
*Nyemba (2022)*	198	Culture	*Chlamydia trachomatis, Neisseria gonorrhoea*	163(82.3)	76.7%-87.3%	35(17.7)	12.7%-23.3%	8
*Gudza-Mugabe (2020)*	88	PCR & DNA Sequencing	*Bacterial vaginosis*	18(20.5)	12.6%-29.6%	70(79.5)	70.4%-87.4%	7
*Njoku (2018)*	18	Culture	*Group B Streptococcus*	13(72.2)	48.9%-90.9%	5(27.8)	9.1%-51.1%	8
*Price (2021)*	221	Metagenomic Sequencing	*Bacterial vaginosis*	85(38.7)	32.1%-45.0%	136(61.5)	55.0%-67.9%	8
*Biobaku (2017)*	36	Culture	*Group B Streptococcus*	13(36.1)	21.1%-52.6%	23(63.9)	47.4%-78.9%	10
*Smullin (2020)*	35	Culture	*Mycoplasma genitalium*	22(62.9)	46.1%-78.2%	13(37.1)	21.8%-53.9%	8
*Short (2020)*	52	DNA Sequencing	*Bacterial vaginosis*	42(80.8)	68.8%-90.5%	10(19.2)	9.5%-31.2%	7
*Makinde (2022)*	8	Culture	*Group B Streptococcus*	4(50.0)	15.3%-84.7%	4(50.0)	15.3%-84.7%	8
*Morgan (2023)*	77	Culture	*Group B Streptococcus*	31(40.3)	29.5%-51.5%	46(59.7)	48.5%-70.5%	7
*Ravindran (2021)*	302	Culture	*Chlamydia trachomatis, Neisseria gonorrhoea*	4(0.01)	0.0%-3.0%	296(99.99)	97.0%-99.7%	8
*Gray (2011)*	390	Culture	*Group B Streptococcus*	77(19.4)	15.9%-23.8%	313(21.7)	76.2%-84.1%	7
*El Beitune (2006)*	35	culture	*Group B streptococcus*	20(19.8%)	40.3%-73.2%	15(14.1%)	26.8%-59.7%	7

**Table 3:** Vaginal colonization bacterial isolate types

Bacterial isolate	No. of studies	Total (N)	Cases	Prevalence, 95% CI
*Group B Streptococcus*(27, 29, 32, 33, 35, 36)	6	3129	632	20.2%. 18.8%-21.6%
*Mycoplasma genitalium*(30)	1	197	35	17.8%, 12.7%-23.4%
*Bacterial vaginosis* (26, 28, 31)	3	794	269	33.9%, 30.6%-37.2%
*Neisseria Gonorrhoea*(25)	1	619	35	5.7%, 4.0%-7.6%
*Chlamydia trachomatis*(25)	1	619	158	25.5%, 22.2%-29.0%
*Escherichia coli*(24)	1	320	52	16.2%, 12.4%-20.5%
*Any STI in pregnancy*(25, 30)	2	816	311	38.1%, 34.8%-41.5%
*Chlamydia trachomatis & Neisseria gonorrhoea*(34)	1	1244	302	24.3%, 21.9%-26.7%

## Data Availability

The data supporting this study’s findings are available in the supplementary material of this article (Supplementary Data S2: study database).

## Abbreviations

AMR: Antimicrobial Resistance
AMSTAR: Assessing the Methodological Quality of Systematic Reviews
ANC: Antenatal Care
CINAHL: Cumulative Index to Nursing and Allied Health Literature
CMA: Comprehensive Meta-Analysis
CI: Confidence Interval
HICs: High-Income Countries
HIV: Human Immunodeficiency Virus
IUGR: Intrauterine Growth restrictions
LBW: Low Birth Weight
LMICs: Low-Middle Income Countries
MESH: Medical Subject Headings
OR: Odds ratio
NIH: National Institutes of Health
NOS: New Castle-Ottawa Scale
PCR: Polymerase Chain Reaction
PRISMA: Preferred Reporting Items for Systematic Reviews and Meta-Analyses
SGA: Small for Gestation
SD: Standard Deviation
SSA: Sub-Saharan Africa

## References

[1] Tumuhamye J, Steinsland H, Tumwine JK, Namugga O, Mukunya D, Bwanga F, Vaginal colonisation of women in labour with potentially pathogenic bacteria: a cross sectional study at three primary health care facilities in Central Uganda. BMC Infectious Diseases. 2020;20(1):98. 32005177 10.1186/s12879-020-4821-6 PMC6995194

[2] Sweeney EL, Kallapur SG, Gisslen T, Lambers DS, Chougnet CA, Stephenson S-A, Placental infection with Ureaplasma species is associated with histologic chorioamnionitis and adverse outcomes in moderately preterm and late-preterm infants. The Journal of Infectious Diseases. 2016;213(8):1340–7. 26671889 10.1093/infdis/jiv587 PMC4799668

[3] Juliana NC, Peters RP, Al-Nasiry S, Budding AE, Morré SA, Ambrosino E. Composition of the vaginal microbiota during pregnancy in women living in sub-Saharan Africa: a PRISMA-compliant review. BMC pregnancy and childbirth. 2021;21(1):1–15. 34479485 10.1186/s12884-021-04072-1 PMC8418042

[4] Abdool Karim SS, Baxter C, Passmore JAS, McKinnon LR, Williams BL. The genital tract and rectal microbiomes: their role in HIV susceptibility and preven on in women. Journal of the International AIDS Society. 2019;22(5):e25300. 31144462 10.1002/jia2.25300 PMC6541743

[5] Mitima KT, Ntamako S, Birindwa AM, Mukanire N, Kivukuto JM, Tsongo K, Prevalence of colonization by Streptococcus agalactiae among pregnant women in Bukavu, Democratic Republic of the Congo. The Journal of Infection in Developing Countries. 2014;8(09):1195–200. 25212085 10.3855/jidc.5030

[6] Ernest AI, Ndaboine E, Massinde A, Kihunrwa A, Mshana S. Maternal vaginorectal colonization by Group B Streptococcus and Listeria monocytogenes and its risk factors among pregnant women a ending tertiary hospital in Mwanza, Tanzania. Tanzania Journal of Health Research. 2015;17(2).

[7] Shah M, Aziz N, Leva N, Cohan D. Group B Streptococcus colonization by HIV status in pregnant women: prevalence and risk factors. Journal of women’s health. 2011;20(11):1737–41. 10.1089/jwh.2011.2888 22011210

[8] Dauby N, Adler C, Miendje Deyi VY, Sacheli R, Busson L, Chamekh M, , editors. Prevalence, risk factors, and serotype distribution of group B Streptococcus colonization in HIV-infected pregnant women living in Belgium: a prospective cohort study. Open Forum Infectious Diseases; 2018: Oxford University Press US. 10.1093/ofid/ofy320 PMC6306564 30619909

[9] Fiseha SB, Jara GM, Woldetsadik EA, Bekele FB, Ali MM. Colonization Rate of Potential Neonatal Disease-Causing Bacteria, Associated Factors, and Antimicrobial Susceptibility Profile Among Pregnant Women A ending Government Hospitals in Hawassa, Ethiopia. Infection and Drug Resistance. 2021;14:3159. 34429615 10.2147/IDR.S326200 PMC8374838

[10] Kamgobe E, Grote S, Mushi MF, Wilson D, Gandye L, Bader O, Multi-drug resistant facultative pathogenic bacteria colonizing the vagina of pregnant women with premature rupture of membrane, Tanzania. East Africa Science. 2020;2(1):29–35.

[11] de Go au MC, Lager S, Sovio U, Gaccioli F, Cook E, Peacock SJ, Human placenta has no microbiome but can contain potential pathogens. Nature. 2019;572(7769):329–34. 31367035 10.1038/s41586-019-1451-5 PMC6697540

[12] Waldorf KMA, McAdams RM. Influence of infection during pregnancy on fetal development. Reproduction. 2013;146(5):R151–R62. 23884862 10.1530/REP-13-0232 PMC4060827

[13] Charlier C, Disson O, Lecuit M. Maternal-neonatal listeriosis. Virulence. 2020;11(1):391–7. 32363991 10.1080/21505594.2020.1759287 PMC7199740

[14] Tumuhamye J, Steinsland H, Bwanga F, Tumwine JK, Ndeezi G, Mukunya D, Vaginal colonization with antimicrobial-resistant bacteria among women in labor in central Uganda: prevalence and associated factors. An microbial Resistance & Infection Control. 2021;10(1):1–11. 10.1186/s13756-021-00897-9 PMC7887551 33597029

[15] Wang P, Tong J-j, Ma X-h, Song F-l, Fan L, Guo C-m, Serotypes, an bio c susceptibilities, and multi-locus sequence type profiles of Streptococcus agalactiae isolates circulating in Beijing, China. PLoS One. 2015;10(3):e0120035. 25781346 10.1371/journal.pone.0120035 PMC4363692

[16] Ozim CO, Mahendran R, Amalan M, Puthussery S. Prevalence of human immunodeficiency virus (HIV) among pregnant women in Nigeria: a systematic review and meta-analysis. 2023;13(3):e050164. 10.1136/bmjopen-2021-050164 PMC9980359 36858473

[17] Manyahi J, Jullu BS, Abuya MI, Juma J, Ndayongeje J, Kilama B, Prevalence of HIV and syphilis infections among pregnant women a ending antenatal clinics in Tanzania, 2011. BMC Public Health. 2015;15(1):501. 25994129 10.1186/s12889-015-1848-5 PMC4492104

[18] Okwaraji YB, Bradley E, Ohuma EO, Yargawa J, Suarez-Idueta L, Requejo J, National routine data for low birthweight and preterm births: Systematic data quality assessment for United Nations member states (2000–2020). BJOG: An International Journal of Obstetrics & Gynaecology. 2023. 10.1111/1471-0528.17699 37932234

[19] Ohuma EO, Moller A-B, Bradley E, Chakwera S, Hussain-Alkhateeb L, Lewin A, National, regional, and global estimates of preterm birth in 2020, with trends from 2010: a systematic analysis. The Lancet. 2023;402(10409):1261–71. 10.1016/S0140-6736(23)00878-4 37805217

[20] Page MJ, McKenzie JE, Bossuyt PM, Boutron I, Ho mann TC, Mulrow CD, The PRISMA 2020 statement: an updated guideline for reporting systematic reviews. International journal of surgery. 2021;88:105906. 33789826 10.1016/j.ijsu.2021.105906

[21] Schiavo JH. PROSPERO: an international register of systematic review protocols. Medical reference services quarterly. 2019;38(2):171–80. 31173570 10.1080/02763869.2019.1588072

[22] Wells GA SB, O’Connell D, Peterson J, Welch V, Losos M, The Newcastle-Ottawa Scale (NOS) for assessing the quality of nonrandomised studies in meta-analyses. 2014.

[23] Thompson DM, Zhao YD. Choosing statistical models to assess biological interaction as a departure from additivity of effects. arXiv preprint arXiv:230103349. 2023.

[24] Madrid L, Maculuve SA, Vilajeliu A, Sáez E, Massora S, Cossa A, Maternal carriage of group B Streptococcus and Escherichia coli in a district hospital in Mozambique. The Pediatric infectious disease journal. 2018;37(11):1145–53. 30312265 10.1097/INF.0000000000001979

[25] Nyemba DC, Peters RP, Medina-Marino A, Klausner JD, Ngwepe P, Myer L, Impact of aetiological screening of sexually transmitted infections during pregnancy on pregnancy outcomes in South Africa. BMC Pregnancy and Childbirth. 2022;22(1):194. 35264142 10.1186/s12884-022-04520-6 PMC8908701

[26] Gudza-Mugabe M, Havyarimana E, Jaumdally S, Garson KL, Lennard K, Tarupiwa A, Human immunodeficiency virus infection is associated with preterm delivery independent of vaginal microbiota in pregnant African women. The Journal of Infectious Diseases. 2020;221(7):1194–203. 31722395 10.1093/infdis/jiz584 PMC7075414

[27] Njoku C, Emechebe C, Agbakwuru A. prevalence and determinants of anogenital colonization by Group B Streptococcus infection among HIV positive and negative women in Calabar, Nigeria. Int J Women’s Health Reprod Sci. 2017;6(1):11–7.

[28] Price JT, Vwalika B, France M, Ravel J, Ma B, Mwape H, HIV-associated vaginal microbiome and inflammation predict spontaneous preterm birth in Zambia. Scientific Reports. 2022;12(1):8573. 35595739 10.1038/s41598-022-12424-w PMC9123167

[29] Biobaku Oluwafunmilola R, Olaleye Atinuke O, Adefusi Olorunwa F, Adeyemi Babalola A, Onipede Anthony O, Loto Olabisi M, Group B streptococcus colonization and HIV in pregnancy: A cohort study in Nigeria. Journal of Neonatal-Perinatal Medicine. 2017;10(1):91–7. 28304326 10.3233/NPM-1685

[30] Smullin CP, Green H, Peters R, Nyemba D, Qayiya Y, Myer L, Prevalence and incidence of Mycoplasma genitalium in a cohort of HIV-infected and HIV-uninfected pregnant women in Cape Town, South Africa. Sexually transmi ed infections. 2020;96(7):501–8. 10.1136/sextrans-2019-054255 PMC7354884 31932358

[31] Short C-ES, Brown RG, Quinlan R, Lee YS, Smith A, Marchesi JR, Lactobacillus-depleted vaginal microbiota in pregnant women living with HIV-1 infection are associated with increased local inflammation and preterm birth. Frontiers in Cellular and Infection Microbiology. 2021;10:596917. 33643930 10.3389/fcimb.2020.596917 PMC7905210

[32] Makinde O, Okusanya B, Osanyin G. Group B Streptococcus vaginal colonization in pregnant women living with HIV infection: prevalence and antibiotic susceptibility at HIV referral centers in Lagos, Nigeria. The Journal of Maternal-Fetal & Neonatal Medicine. 2022;35(25):9098–104. 34894995 10.1080/14767058.2021.2015575

[33] Morgan JA, Hankins ME, Callais NA, Albritton CW, Vanchiere JA, Betcher RE, Group B Streptococcus rectovaginal colonization and resistance patterns in HIV-positive compared to HIV-negative pregnant patients. American Journal of Perinatology. 2023;40(14):1573–8. 34784616 10.1055/s-0041-1739356

[34] Ravindran J, Richardson BA, Kinuthia J, Unger JA, Drake AL, Osborn L, Chlamydia, gonorrhea, and incident HIV infection during pregnancy predict preterm birth despite treatment. The Journal of 511 infectious diseases. 2021;224(12):2085–93. 10.1093/infdis/jiab277 PMC8672741 34023871

[35] Gray KJ, Kafulafula G, Matemba M, Kamdolozi M, Membe G, French N. Group B Streptococcus and HIV infection in pregnant women, Malawi, 2008–2010. Emerging infectious diseases. 2011;17(10):1932. 22000375 10.3201/eid1710.102008 PMC3310663

[36] El Beitune P, Duarte G, Maffei CML, Quintana SM, Rosa ACJDS, Silva E, Group B Streptococcus carriers among HIV-1 infected pregnant women: prevalence and risk factors. European Journal of Obstetrics & Gynecology and Reproductive Biology. 2006;128(1–2):54–8. 16621230 10.1016/j.ejogrb.2006.02.017

[37] Benedetto C, Tibaldi C, Marozio L, Marini S, Masuelli G, Pelissetto S, Cervicovaginal infections during pregnancy: epidemiological and microbiological aspects. The Journal of Maternal-Fetal & Neonatal Medicine. 2004;16(2):9–12. 10.1080/14767050410001727107 15590426

[38] Foessleitner P, Petricevic L, Boerger I, Steiner I, Kiss H, Rieger A, HIV infection as a risk factor for vaginal dysbiosis, bacterial vaginosis, and candidosis in pregnancy: A matched case-control study. Birth. 2021;48(1):139–46. 33462893 10.1111/birt.12526 PMC8247846

[39] Govender S, Theron G, Odendaal H, Chalkley L. Prevalence of genital mycoplasmas, ureaplasmas and chlamydia in pregnancy. Journal of Obstetrics and Gynaecology. 2009;29(8):698–701. 19821660 10.3109/01443610903184033

[40] Zenebe A, Eshetu B, Gebremedhin S. Association between maternal HIV infection and birthweight in a tertiary hospital in southern Ethiopia: retrospective cohort study. Italian Journal of Pediatrics. 2020;46:1–9. 32448252 10.1186/s13052-020-00834-3 PMC7247191

[41] Joachim A, Matee MI, Massawe FA, Lyamuya EF. Maternal and neonatal colonisation of group B streptococcus at Muhimbili National Hospital in Dar es Salaam, Tanzania: prevalence, risk factors and antimicrobial resistance. BMC public Health. 2009;9:1–7. 19948075 10.1186/1471-2458-9-437 PMC2791767

[42] Seale AC, Koech AC, Sheppard AE, Barsosio HC, Langat J, Anyango E, Maternal colonization with Streptococcus agalactiae and associated stillbirth and neonatal disease in coastal Kenya. Nature microbiology. 2016;1(7):1–10. 10.1038/nmicrobiol.2016.67 PMC4936517 27572968

[43] Temmerman M, Plummer FA, Mirza NB, Ndinya-Achola JO, Wamola IA, Nagelkerke N, Infection with HIV as a risk factor for adverse obstetrical outcome. Aids. 1990;4(11):1087–94. 2282181 10.1097/00002030-199011000-00006

[44] Marando R, Seni J, Mirambo MM, Falgenhauer L, Moremi N, Mushi MF, Predictors of the extended-spectrum-beta lactamases producing Enterobacteriaceae neonatal sepsis at a tertiary hospital, Tanzania. International Journal of Medical Microbiology. 2018;308(7):803–11. 29980372 10.1016/j.ijmm.2018.06.012 PMC6171784

[45] Kovacs D, Silago V, Msanga DR, Mshana SE, Seni J, Oravcova K, The hospital environment versus carriage: Transmission pathways for third-generation cephalosporin-resistant bacteria in blood in neonates in a low-resource country healthcare setting. Scientific Reports. 2022;12(1):8347. 35589773 10.1038/s41598-022-11626-6 PMC9120020

[46] Xiao P-L, Zhou Y-B, Chen Y, Yang M-X, Song X-X, Shi Y, Association between maternal HIV infection and low birth weight and prematurity: a meta-analysis of cohort studies. BMC pregnancy and childbirth. 2015;15:1–11. 26450602 10.1186/s12884-015-0684-z PMC4599647

[47] Cools P, van de Wijgert JH, Jespers V, Crucitti T, Sanders EJ, Verstraelen H, Role of HIV exposure and infection in relation to neonatal GBS disease and rectovaginal GBS carriage: a systematic review and meta-analysis. Scientific Reports. 2017;7(1):13820. 29062060 10.1038/s41598-017-13218-1 PMC5653843

[48] Olaru ID, Tacconelli E, Yeung S, Ferrand RA, Stabler RA, Hopkins H, The association between an microbial resistance and HIV infection: a systematic review and meta-analysis. Clinical Microbiology and Infection. 2021;27(6):846–53. 33813126 10.1016/j.cmi.2021.03.026

